# Risk assessment of venous thromboembolism and thromboprophylaxis in pregnant women hospitalized with cancer: Preliminary results from a risk score

**DOI:** 10.6061/clinics/2018/e368

**Published:** 2018-10-05

**Authors:** Eliane Azeka Hase, Venina Isabel Poço Viana Leme de Barros, Ana Maria Kondo Igai, Rossana Pulcinelli Vieira Francisco, Marcelo Zugaib

**Affiliations:** Departamento de Obstetricia e Ginecologia, Faculdade de Medicina FMUSP, Universidade de Sao Paulo, Sao Paulo, BR

**Keywords:** Cancer, Hospitalization, Pregnancy, Risk Assessment, Venous Thrombosis

## Abstract

**OBJECTIVES:**

Hospitalized patients with cancer are at high risk of developing venous thromboembolism, and the risk increases with pregnancy. The aim of this study was to apply a thromboprophylaxis protocol with a venous thromboembolism risk score for hospitalized pregnant women with cancer and to evaluate the effects on maternal morbidity and mortality.

**METHODS:**

A longitudinal and prospective study was conducted from December 2014 to July 2016. The venous thromboembolism risk score was modified from the guidelines of the Royal College of Obstetricians and Gynaecologists. Patients were classified as low (score <3) or high risk (score ≥3). The high-risk group received thromboprophylaxis with low-molecular-weight heparin, unless the patient had a contraindication for anticoagulation. One patient could have undergone more than one evaluation.

**RESULTS:**

Fifty-two ratings were descriptively analyzed: 34 (65.4%) were classified as high risk, and 28/34 (82.3%) received low-molecular-weight heparin, 1 received unfractionated heparin, and 5 did not receive intervention. Most patients (23/52; 44.2%) had breast cancer. The main risk factors for venous thromboembolism in the high-risk group were chemotherapy (within 6 months; 22/34; 64.7%). No patient exhibited venous thromboembolism, adverse effects of anticoagulation or death up to three months after hospitalization.

**CONCLUSIONS:**

Most pregnant women with cancer had a high risk for venous thromboembolism at the time of hospitalization. Breast cancer was the most prevalent cancer, and recent chemotherapy was the main risk factor for anticoagulation. The application of a thromboprophylaxis protocol and determination of a venous thromboembolism risk score for these patients was useful for the prevention of maternal morbidity and mortality due to venous thromboembolism.

## INTRODUCTION

The diagnosis of cancer during pregnancy is a rare event. However, its incidence has increased in recent years, mainly because these days, women often delay childbearing until after the age of 30, which coincides with the time that cancers tend to appear 1,2. Studies have shown that cancer patients are at high risk for developing venous thromboembolism (VTE), especially when hospitalized 3, and this risk is even greater when associated with pregnancy because pregnancy also increases the risk of thrombosis 4-6. Thromboembolic events are among the leading causes of maternal morbidity and mortality during pregnancy and the postpartum period and are the leading causes of maternal death in developed countries 7-9. Other risk factors for VTE include metastatic disease 10, rapidly growing tumors 11, and biologically aggressive cancers associated with a poor prognosis 12.

To our knowledge, no studies in the literature have assessed the risk of VTE in hospitalized pregnant women diagnosed with cancer who undergo thromboprophylaxis. Therefore, this study was conducted to evaluate the application of a thromboprophylaxis protocol and a VTE risk score for pregnant women who were hospitalized with a diagnosis of cancer during clinical and/or surgical treatment at our Obstetrics clinic and who were followed up to 3 months after hospitalization.

## MATERIALS AND METHODS

This was a longitudinal and prospective study of pregnant women diagnosed with cancer who were admitted to the Obstetrics Clinic of the Hospital das Clínicas da Faculdade de Medicina da Universidade de São Paulo (HC-FMUSP) for clinical and/or surgical treatment between December 1, 2014, and July 31, 2016, and followed up to 3 months after hospitalization. The study aimed to evaluate the application of a thromboprophylaxis protocol and a VTE risk score. The analysis reported here was preliminary and consisted of partial results of a trial involving other diseases including cancer, which is being conducted at HC-FMUSP and is registered at Clinicaltrials.gov 13.

### Patient recruitment

**Figure 1:**
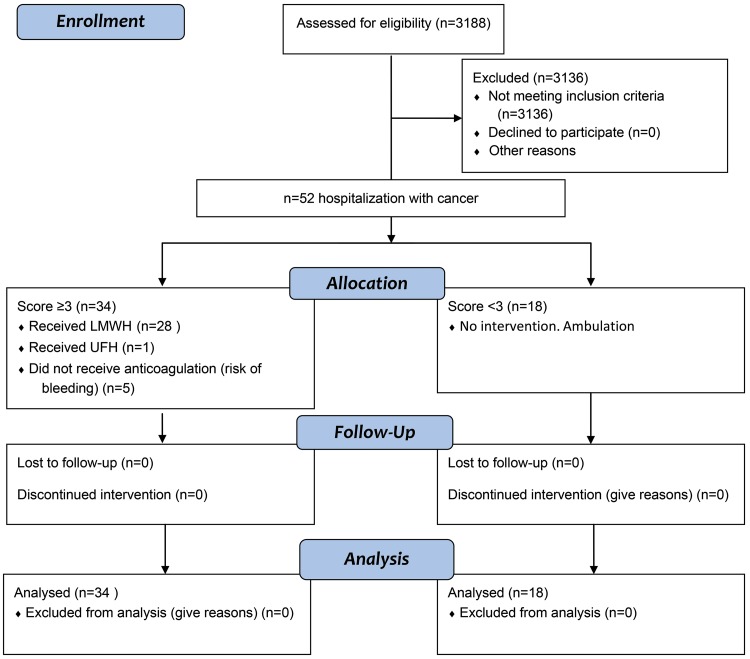
Consort flow diagram.

We have developed an electronic formula (Table 1), attached to the electronic patient records. The data are described in Table 1 and were collected during hospitalization. The formula was applied to all pregnant women admitted for clinical and/or surgical treatment, and in this study, we included and analyzed only patients with a diagnosis of cancer during pregnancy (Figure 1).

### Risk score

The VTE risk score used was developed and modified based on the risk score of the Royal College of Obstetricians and Gynaecologists 14. The VTE risk score used to assess patients was an addition system that was based on the risk factors of the patients; the patients had different risk factors (Table 1) that were classified as high (3 points), moderate (2 points) or low risk factors (1 point), and the VTE risk score was calculated by summing the points of these risk factors. The patients were classified as low risk for VTE (score <3) or high risk for VTE (score ≥3). Table 1 shows how each risk factor contributes points to the score. The formula was applied at clinical hospitalization and repeated after 7 days of hospitalization and after delivery. One patient could undergo more than one evaluation for the risk score for VTE, depending on the number of admissions she had during the period proposed in this study.

### Thromboprophylaxis treatment

The high-risk group of VTE patients (score ≥3) received thromboprophylaxis with enoxaparin unless a contraindication for anticoagulation, such as active bleeding or a high bleeding risk, was present (Table 1). Alternative methods were also offered to patients, such as ambulation and/or bandaging of the limbs, especially for patients with contraindications for anticoagulation (Table 1). Anticoagulation was started eight hours after delivery or surgery. If the patient was hospitalized for clinical treatment, anticoagulation was started soon after the patient was in the ward. Enoxaparin was administered once a day, subcutaneously. The dose depended on the weight of the patient 14: patients <50 kg received enoxaparin 20 mg/day; patients ≥50-<90 kg received enoxaparin 40 mg/day; patients ≥90 to <130 kg received enoxaparin 60 mg/day; patients ≥130 to <170 kg received enoxaparin 80 mg/day; and patients ≥170 kg received enoxaparin 0.6 mg/kg/day. Anticoagulation was maintained during the entire hospitalization. After discharge, for patients with scores ≥3 for VTE and a high risk factor (Table 1), anticoagulation was maintained for 40 days. The patients with scores ≥3 for VTE without a high risk factor were prescribed anticoagulation treatment for 15 days. Doubts about the intervention or adverse events were reported to the investigators. The authors checked daily whether there were errors in the filling of the formula and whether the dose was correct and monitored whether there was any change in the patients' clinical conditions. After discharge, the patients were asked to return to the ambulatory clinic for evaluation, depending on their clinical conditions, after 7 to 15 days. Additionally, 3 months after discharge, the patients received a phone call from a nurse and were asked whether they had any medical complications and a need for rehospitalization. If the patient had a medical complication, she was asked about the kind of complication.

### Statistical analyses

To examine the characteristics of the pregnant women with cancer, a descriptive analysis of the data was performed. The mean, standard deviation (SD), and minimum and maximum values were considered to present quantitative variables. For the qualitative variables, frequencies, percentages and contingency tables were used to understand the data.

The association between qualitative variables was evaluated using the Chi-square test or Fisher's exact test, and for comparisons of groups in relation to quantitative variables, Student's t-test was used. The software used was IBM SPSS Statistics for Windows, Version 20.0. The significance level was 5%; that is, *p* values lower than 0.05 were set as the threshold for significance.

### Ethics

This study followed the tenets of the Declaration of Helsinki and the rules of Resolution No. 196/96 of the Brazilian National Health Council. All patients were informed of the research objectives. Only those who voluntarily accepted and signed informed consent forms to participate in the study who were approved by the local committee of number CAAE 37431414.9.0000.0068 were included.

## RESULTS

During the study period, 3188 (2671 patients) VTE score ratings were performed at the Obstetrics unit, 52 of which were ratings of cancer (34 patients) that were included in our analysis (Figure 1). The mean age was 31 years (17-46 years): 15 ratings (28.8%) were for patients ≥35 years, 5 ratings (9.6%) were for patients ≥40 years, 10 ratings (19.2%) were for patients 35-40 years old, and 37 ratings (71.2%) were for patients <35 years (Table 3). The age of the patients was significantly different in both the high- and low-risk groups (*p*=0.002; Table 2). The BMI was not significantly different between the VTE groups (Table 2). The weight ranged from 46 kg to 99 kg in the total cohort.

Ten (19.2%) of the 52 ratings were obtained from patients who had undergone cancer surgery during gestation. Fourteen (26.9%) of the 52 ratings were from patients who had metastases (Table 2). There were no significant differences between the VTE groups for either of these variables.

There were a total of 20/52 (38.5%) ratings performed due to hospitalization for clinical or surgical cancer treatment: 9 (31%) after vaginal delivery and 20 (69%) after C-section. There were no significant differences in terms of these characteristics between the VTE groups (Table 2).

Of the 52 ratings performed, 18 were classified as low risk for VTE (14 patients), and 34 (65.4%) were classified as high risk for VTE (23 patients) (Tables 2 and 3). Of these 34 high-risk cases, 28 (82.3%) received LMWH (enoxaparin), 1 case received unfractionated heparin (UFH) in a prophylactic dose, and 5 (14.7%) cases had contraindications to medication due to the risk of bleeding and therefore used alternative methods, such as ambulation (1 case of leukemia, 3 cases of cervical cancer, 1 case of bone cancer). The doses of enoxaparin ranged from 40-60 mg: enoxaparin 40 mg was indicated for 25 cases, and enoxaparin 60 mg was indicated for 3 cases. Only one patient received UFH. She was a patient with lymphoma that caused spinal cord compression, and she was bedridden and could go into labor at any time. Therefore, it was preferable to use UFH because of the risk of bleeding using enoxaparin. One patient classified as low risk for VTE (score 2) received enoxaparin because she had soft tissue sarcoma in the right leg, which made ambulation difficult, and this limitation was considered to possibly increase the risk of VTE.

The risk factors found in the high-risk group for VTE (score ≥3) are shown in Table 3 and included chemotherapy during the previous 6 months (22/34; 64.7%), ≥40 years of age (5/34; 14.7%), ≥35 to <40 years of age (9/34; 26.5%), multiparity (≥3 deliveries) (7/34; 20.6%), cancer surgery during pregnancy/postpartum (9/34; 26.5%) and infection (4/34; 11.8%). Chemotherapy was the most significantly prevalent risk factor for VTE in the high-risk group for VTE compared to the low-risk group (*p*<0.001), and patients <35 years of age were most prevalent in the low-risk group for VTE (*p*=0.029).

The types of cancer in the high-risk group were as follows (Table 4): breast cancer, 19 (55.9%); leukemia, 4 (11.8%); cervical cancer, 4 (11.8%); lymphoma, 2 (5.9%); gastrointestinal cancer, 2 (5.9%); bone cancer, 2 (5.9%); and ovarian cancer, 1 (2.9%). The low-risk group had significantly fewer breast cancers (4/18; 22.2%) than the high-risk group (*p*=0.038). There were two terminations of pregnancies in the first trimester and one at 18 weeks gestation in the low-risk group, due to risk to the mother's life (Table 4).

No patient exhibited VTE, adverse effects of anticoagulation, or death up to three months after hospitalization and delivery in either the high-risk or low-risk group.

## DISCUSSION

In recent decades, there has been an increase in the incidence of cancer diagnoses in the general population, which may be due to the aging of the population. Currently, women are frequently postponing the age of first pregnancy, which likely contributes to the incidence of cancer diagnoses during pregnancy 15. Our results showed that 15 ratings of cancer patients (28.8%) were for patients aged 35 years or older. The hypothesis that the incidence of cancer is also increasing in younger patients is demonstrated by our finding that 37 ratings (71.2%) were for patients younger than 35 years. Cancer is the second most common cause of death in women during the reproductive years, and breast cancer is one of the most commonly diagnosed cancers in women younger than 35 years 16. Breast cancer is the most frequent cancer found in women in the city of São Paulo 17,18. Of 52 ratings in our study, 23 (44.2%) were for patients hospitalized with breast cancer, and 19 (36.5%) of these ratings had a high risk score for VTE. Of the 23 ratings of breast cancer, 12 (52.2%) were for patients younger than 35 years, and 5 (21.7%) were for patients greater than or equal to 40 years of age.

Pregnancy alone is the most thrombogenic stage of a woman's life 19.

VTE is a complication commonly linked to active cancers and is furthermore exacerbated by associated treatments 20. In a population-based study, cancer was associated with a 4.1-fold greater risk of thrombosis, whereas the use of chemotherapy increased the risk 6.5-fold 21,22. In our series, chemotherapy in the past 6 months was the most significant risk factor in the high-risk group and was performed in 64.7% of these cases. VTE remains the major cause of morbidity and mortality in hospitalized patients and is the second leading cause of death in patients with overt malignant disease 23,24.

Patients with active cancer face a very high risk of developing VTE postoperatively. In the absence of thromboprophylaxis, the overall incidence of postoperative deep-vein thrombosis (DVT) is approximately two times higher in patients with cancer than in patients without malignant disease 25,26. According to Geerts et al. 27, cancer patients who undergo surgery have a 2-fold increased risk of developing postoperative DVT compared to non-cancer patients and are three times more likely to have a fatal pulmonary embolism. In the present study, 10/52 ratings (19.2%) were for patients who underwent surgery during pregnancy, and surgery was considered an independent risk factor (1 point - Table 1). No patient in our study presented VTE postoperatively, but 6/10 (60%) patients with ratings of cancer surgery during pregnancy had a high risk score for VTE (Table 2).

The most frequently reported cancers during pregnancy are breast cancer, hematological cancers, cervical cancer, and malignant melanoma 15. This was consistent with our study, in which most of the ratings were for breast cancer (23/52; 44.2%), followed by hematological cancers (9/52; 17.3%), cervical cancer (9/52; 17.3%), and others (11/52; 21.1%), including ovarian cancer (2), soft tissue sarcoma (2), bone cancer (2), gastrointestinal cancers (2), carcinoid tumor (1), thyroid cancer (1) and adrenal cancer (1). A retrospective study with a total of 2826 pregnant women with underlying malignancies concluded that the risk of VTE was high for patients with cervical cancer (OR 8.64, 95% CI (2.15–34.79)), ovarian cancer (OR 10.35, 95% CI (1.44–74.19)), Hodgkin's disease (OR 7.87, 95% CI (2.94–21.05)) and myeloid leukemia (OR 20.75, 95% CI (6.61–65.12)) 28.

Despite the known increased risk of an initial VTE and recurrences in cancer patients, along with the associated costs, few analyses have been performed on the economic impact of VTE in this patient population 29. Improving the implementation guidelines in patients with VTE and cancer is of utmost importance to reduce the risk of recurrence, as well as hospital stay durations 20 and their associated costs. VTE is an independent prognostic factor and is a largely preventable disease when thromboprophylaxis is appropriately used, as recommended worldwide by International Clinical Practice 22,24. This study was conducted due to the lack of data in the literature regarding pregnant women hospitalized with cancer who were assigned a specific score for thromboembolic risk. Of the 52 ratings performed in our series, 34 (65.4%) were classified as high risk for VTE (23 patients). These patients could benefit from thromboprophylaxis.

The American Society of Clinical Oncology Clinical Practice, Guideline reports that cancer patients undergoing surgery and hospitalization for acute medical illness or with reduced mobility should benefit from thromboprophylaxis, in the absence of bleeding or other contraindications to anticoagulants. Thromboprophylaxis in cancer outpatients receiving systemic therapies is still being debated 3. Numerous strategies have been developed to improve VTE prophylaxis practices in cancer patients as well as to elucidate the best appropriate anticoagulant regimen for each individual cancer patient. The National Comprehensive Cancer Center guidelines for the management of VTE in cancer patients consider pregnancy as an isolated risk factor for VTE in cancer patients 30.

To our knowledge, this is the only prospective study of thromboprophylaxis in hospitalized pregnant women with cancer, and we did not observe VTE during hospitalization or at three months after hospital delivery. This finding demonstrates the possibility of using a thromboprophylaxis risk score for pregnant women hospitalized with a diagnosis of cancer who undergo clinical and/or surgical treatment. Cancer is a rare disease during pregnancy, and therefore, the data in the literature are scarce. This is a limitation of our study because it does not allow comparisons with our results. Further studies are required to contribute to the evaluation of thromboprophylaxis for VTE in hospitalized pregnant women with cancer.

### Conclusion

Most pregnant women with cancer had a high risk for VTE at the time of hospitalization. In our study, breast cancer was the most prevalent type of cancer. The use of chemotherapy in the previous 6 months was the main risk factor for anticoagulation. In this study sample, we did not observe VTE during hospitalization or at three months after hospital delivery. The application of a thromboprophylaxis protocol and determination of a VTE risk score for pregnant women with a cancer diagnosis who were hospitalized for clinical and/or surgical treatment was useful for the prevention of maternal morbidity and mortality due to VTE.

## AUTHOR CONTRIBUTIONS

Hase EA is the principal investigator, who provided substantial contributions to the conception and design of the study, and was responsible for the data acquisition, analysis and interpretation; manuscript draft; critical revision of the manuscript for important intellectual content; and final approval of the version to be published. Barros VI also provided substantial contributions to the conception and design of the study; was responsible for the data acquisition, analysis and interpretation; manuscript draft, critical revision of the manuscript for important intellectual content; and final approval of the version to be published. Igai AM provided substantial contributions to the conception or design of the study and was responsible for the data interpretation, critical revision of the manuscript for important intellectual content and final approval of the version to be published. Francisco RP was responsible for the data interpretation, critical revision of the manuscript for important intellectual content and final approval of the version to be published. Zugaib M was responsible for the analysis of data, revision of the manuscript for important intellectual content and final approval of the version to be published.

## Figures and Tables

**Table 1:** VTE risk score for hospitalized pregnant women (HC-FMUSP, 2016). Score ≥3 indicate thromboprophylaxis.

High risk factors (3 points)	Moderate risk factors (2 points)	Low risk factors (1 point)
**Previous VTE** • Recurrent • During gestation or after delivery • Linked to the use of hormones • No triggering factor **High-risk thrombophilia** • Homozygous factor V Leiden • Homozygous mutant prothrombin antithrombin deficiency • Thrombophilia association • Antiphospholipid syndrome (AFS) **Cardiopathies** • Mechanical valve prostheses • Atrial fibrillation or flutter • Cyanotic cardiopathies • Intracavitary thrombosis • Severe ventricular dysfunction • Severe dilation of heart cameras • Pulmonary hypertension	**• Previous VTE associated with a triggering factor** **Thrombophilia** • Homocysteine >15 mmol/l • Heterozygous Leiden factor • Heterozygous mutant prothrombin • Protein C deficiency • Protein S deficiency • Suspected AFS **Clinical conditions** • Cancer (in the previous 6 months) • Chemotherapy (within 6 months) • Cyanotic pneumopathy • Paraplegia • Pyelonephritis/pneumonia/ puerperal infection • Puerperal hemorrhage > 1000 ml • Age ≥40 years • BMI ≥40 kg/m^2^ • Immobility in bed more than 4 days before caesarean section	• Dehydration • Smoker (>20 cig/day) • Multiple pregnancies • Hyperemesis • Age ≥35 y • Parity ≥3 deliveries • Any surgical procedure in the gestation or puerperium • Gross varicose veins
**Other systemic diseases:** • Nephrotic proteinuria (≥3.5 G/24 hours prior to gestation or during the first trimester) • Sickle-cell anemia • Systemic lupus erythematosus* • Acute rheumatological disease* • Intestinal inflammatory disease* • Malignant neoplasmsDigestive tract tumor (pancreas and stomach)Lung tumor **Morbidity in previous gestation with positive thrombophilia (genetic and/or acquired)** • Previous stillbirth without malformations • Placental abruption • Severe placental impairment: • Zero or reverse diastole in the umbilical artery • Restricted fetal growth (*p*<3) • Oligoamnios **Immobility in bed for longer than one week with BMI ≥30 kg/m^2^**		**Risk of bleeding** **Preferably using mechanical methods** • *Active bleeding* • *Active peptic ulcers* • *Uncontrolled systemic arterial hypertension (>180 x 110 mm Hg)* • *Coagulopathy (thrombocytopenia <70,000 or INR >1.5)* • *Renal insufficiency (creatinine >1.5 mg/dl)* • *Cranial or ocular surgery <2 weeks* • *Hepatic/cerebral metastasis* • *Premature rupture of membranes* • *Drugs (concomitant aspirin or NSAIDs)*

*Disease activity requiring hospitalization.

**Table 2:** Demographic and delivery characteristics of pregnant women at low and high risk of venous thromboembolism during hospitalization (HC-FMUSP, 2016).

Characteristic	Score ≥3 n (%)	Score <3 n (%)	Total (%)	*p* value
	n=34 (65.4)	n=18 (34.6)	n=52	
Age, years Media (min-max) ±SD	32.9 (22-46) ± 6.27	27.3 (17-35) ± 4.75	31.04 (17-46) ± 6.33	0.002^a^
BMI, kg/m^2^ Media (min-max) ±SD	27.12 (20-35) ±3.24	27.2 (20-37) ± 4.63	27.15 (20-37) ± 3.73	0.939^a^
**Score information**				
Clinical/surgical treatment*	15 (44.1)	5 (27.8)	20 (38.5)	0.243^b^
Mode of delivery	18	11	29	0.999^c^
*Vaginal delivery*	6 (33.3)	3 (27.3)	9 (31.0)	
*C/S delivery*	12 (66.7)	8 (72.7)	20 (69)	
**Cancer surgery**	9 (26.5)	6 (33.3)	15 (28.8)	0.606^b^
*Pregnancy*	6 (17.6)	4 (22.2)	10 (19.2)	
*Postpartum*	3 (8.8)	2 (11.1)	5 (9.6)	
**Metastatic cancer**	9 (26.5)	5 (27.8)	14 (26.9)	0.999^c^

Score ≥3 = high risk for VTE; Score <3 = low risk for VTE. *Included cancer surgery during and after pregnancy. HC-FMUSP, Hospital das Clínicas da Faculdade de Medicina da Universidade de São Paulo. ^a^ Students t-test; ^b^ Chi-square test; ^c^ Fisher's exact test.

**Table 3:** Risk factors in pregnant patients with cancer and a VTE risk score ≥3 (high risk) or a VTE risk score <3 (HC-FMUSP, 2016).

Risk factors for VTE Total	Score ≥3 n (%) 34	Score <3 n (%) 18	Total n (%) 52	*p* Value
Chemotherapy (within 6 months)	22 (64.7)	2 (11.1)	24 (46.2)	**<0.001^a^**
Age ≥40 years	5 (14.7)	0 (0)	5 (9.6)	0.150^a^
**Age ≥40 years**	5 (14.7)	0 (0)	5 (9.6)	
**Age ≥35 and <40 years**	9 (26.5)	1 (5.6)	10 (19.2)	**0.029^a^**
**Age <35 years**	20 (58.8)	17 (94.4)	37 (71.2)	
Parity ≥3 pregnancies	7 (20.6)	1 (0)	8 (15.4)	0.236^a^
Cancer surgery during pregnancy/postpartum	9 (26.5)	6 (33.3)	15 (28.8)	0.606^b^
Presence of infections	4 (11.8)	0 (0)	4 (7.7)	0.285^a^
**Thromboprophylaxis**				
Enoxaparin	28 (82.3)	1 (5.5)		
Unfractionated heparin	1 (3)	0		
Contraindication	5 (14.7)	0		

VTE, venous thromboembolism; HC-FMUSP, Hospital das Clínicas da Faculdade de Medicina da Universidade de São Paulo. ^a^ Fisher's exact test; ^b^ Chi-square test.

**Table 4:** Type of cancer and risk score for VTE in pregnant patients hospitalized for clinical treatment and/or delivery (HC-FMUSP, 2016).

Type of cancer Total	Score ≥ 3 n (%) 34	Score < 3 n (%) 18	Total n (%) 52	*p* value^c^
Breast	19 (55.9)	4 (22.2)	23 (44.2)	0.04
Leukemia	4 (11.8)	1 (5.5)	5 (9.6)	0.43
Cervix	4 (11.8)	5 (27.8)^b^	9 (17.3)	0.14
Lymphoma	2 (5.9)	2 (11.1)	4 (7.7)	0.43
Gastrointestinal	2 (5.9)	0	2 (3.8)	0.42
Bone	2 (5.9)	0	2 (3.8)	0.42
Ovary	1 (2.9)	1 (5.5)^a^	2 (3.8)	0.57
Carcinoid tumor	0	1 (5.5)	1 (1.9)	0.34
Soft tissue sarcoma	0	2 (11.1)	2 (3.8)	0.11
Thyroid cancer	0	1 (5.5)	1 (1.9)	0.34
Adrenal cancer	0	1 (5.5)^a^	1 (1.9)	0.34

Score ≥3=high risk for VTE; score <3=low risk for VTE. ^a^pregnancy termination; ^b^1 case of pregnancy termination. HC-FMUSP, Hospital das Clínicas da Faculdade de Medicina da Universidade de São Paulo; ^c^Fisher's exact test.
